# Traces of Human-Mediated Selection in the Gene Pool of Red Deer Populations

**DOI:** 10.3390/ani13152525

**Published:** 2023-08-04

**Authors:** Nina Moravčíková, Radovan Kasarda, Radoslav Židek, John Colin McEwan, Rudiger Brauning, Tomás Landete-Castillejos, Louis Chonco, Juraj Ciberej, Jaroslav Pokorádi

**Affiliations:** 1 Faculty of Agrobiology and Food Resources, Slovak University of Agriculture, 949 76 Nitra, Slovakia; radoslav.zidek@uniag.sk; 2 AgResearch, Invermay Agricultural Research Centre, Mosgiel 9024, New Zealand; john.mcewan@agresearch.co.nz (J.C.M.); rudiger.brauning@agresearch.co.nz (R.B.); 3 Instituto de Recursos Cinegéticos-Instituto de Desarrollo Regional, University of Castilla-La Mancha, 02071 Albacete, Spain; tomas.landete@uclm.es (T.L.-C.); louis.chonco@uclm.es (L.C.); 4 Department of Breeding and Diseases of Game, Fish and Bees, Ecology and Cynology, University of Veterinary Medicine and Pharmacy, 041 81 Košice, Slovakia; 5 Slovak Association of Deer Farming, 811 01 Bratislava, Slovakia; jaroslav.pokoradi@gmail.com

**Keywords:** animal breeding, bottleneck, *Cervus elaphus*, diversity, founder effect, genotyping-by-sequencing, gene flow

## Abstract

**Simple Summary:**

Human activities have left indelible traces in the gene pool of livestock as well as recently domesticated wild animal species which are manifested by changes in their genome structure, often accompanied by the significant loss of biodiversity, especially in the case of small local populations. Despite the benefits of deer farming, intensive artificial selection associated with domestication can lead to several negative effects, including founder effects, inbreeding depression, or rapid decline in effective population size, which are also often present in traditional livestock. This study investigates the effect of human-mediated selection on the diversity of seven farmed red deer populations compared to two wild populations through the quantification of the level of genomic heterozygosity, inbreeding, admixture, and gene flow. These results will help to improve traditional breeding schemes and assist in a more sustainable utilisation of available animal genetic resources.

**Abstract:**

In this study, we analysed the effect of human-mediated selection on the gene pool of wild and farmed red deer populations based on genotyping-by-sequencing data. The farmed red deer sample covered populations spread across seven countries and two continents (France, Germany, Hungary, Latvia, New Zealand, Poland, and Slovakia). The Slovak and Spain wild red deer populations (the latter one in a large game estate) were used as control outgroups. The gene flow intensity, relationship and admixture among populations were tested by the Bayesian approach and discriminant analysis of principal components (DAPC). The highest gene diversity (*H_e_* = 0.19) and the lowest genomic inbreeding (*F_HOM_* = 0.04) found in Slovak wild population confirmed our hypothesis that artificial selection accompanied by bottlenecks has led to the increase in overall genomic homozygosity. The Bayesian approach and DAPC consistently identified three separate genetic groups. As expected, the farmed populations were clustered together, while the Slovak and Spanish populations formed two separate clusters. Identified traces of genetic admixture in the gene pool of farmed populations reflected a strong contemporary migration rate between them. This study suggests that even if the history of deer farming has been shorter than traditional livestock species, it may leave significant traces in the genome structure.

## 1. Introduction

The genetic diversity of European red deer populations (*Cervus elaphus*) has been historically affected either by environmental changes or especially by anthropogenic activity [1]. Ludt et al. [2] found that the different subspecies in Western Europe could not be differentiated by assessing variations in mtDNA cytochrome b. Sommer et al. [3] compared genetic and archaeological evidence to conclude that in the last glacial maximum, red deer (and implicitly and non-artic fauna) retracted to habitats in the Iberian peninsula–southern France (western line), shelters in Eastern Europe–Balkans (Eastern line), and a third line likely lived in Italy. Subsequently, Skog et al. [4] examined current and paleontological samples to confirm these three lines, two of which populated northern habitats when the ice melted (western and eastern Europe lines). However, there is also evidence of genetic exchange between animals belonging to these two lines before the last glacial maximum disappeared: in a single mortality event caused by a cave collapse in north-western Spain dated 35,000 years ago, 15 deer died and some belonged to the Balkan line, whereas others belonged to the Iberian line [5]. Although some authors defended the existence of some of the subspecies (specifically, *C. e. hispanicus*) based on nuclear DNA microsatellites as opposed to mitochondrial DNA [6], other studies confirmed using the microsatellites the lines reported by Skog et al. [4,7]. Thus, during these natural migrations and because red deer populations were spread in different habitats, the frequencies of their genes could diverge due to drift, natural selection, and adaptation to given environmental conditions. It follows that the anthropogenic influence will have left significant traces in the gene pool of red deer populations and simultaneously affected their genetic structure [8,9,10].

Red deer belong to a species with probably the most extensive human-assisted geographical translocation over the last 100 years. This has been driven by the desire to improve trophy quality and game park establishment (via clubs and organised hunting) [8,11]. Translocation of non-native red deer genotypes to the local autochthonous populations starting in the early Neolithic age is recognised as the most important human intervention with a serious effect on their genetic structure [8,9]. Such hybridisation between genetically distinct populations could lead to the introgression of non-native and loss of native alleles [12]. Gene flow between populations from distinct habitats could subsequently limit the ability to adapt locally and reduce the short-term “fitness” of autochthonous populations. On the other hand, the increasing level of genetic variability caused by translocation could also positively affect population viability through heterosis or reduction of inbreeding depression depending on the genetic distance of the species (taxon) under hybridisation [13,14,15]. In European red deer populations, such migration events were relatively common, predominantly to restore or strengthen local autochthonous populations to avoid local extinction or transport of trophy animals [11,16,17,18]. Many of such populations were morphologically distinct and assigned as subspecies previous to the studies in the last 15 years [19]. However, as pointed out above, most authors consider the five European subspecies as unique red deer species without subspecies, although with genetic variability among populations or lines, particularly because it takes 90,000–400,000 years for a subspecies to diverge, whereas European populations sheltered in a reduced area in southern Europe up to 12,000 years ago [2,4,7,20]. As a significant comparison, most archaeologists agree that humans arrived in America only during deglaciation, about 15,000 years ago, and nobody considers their native population a different human subspecies [21].

In addition to the translocation of individuals considered of high-genetic quality, human-mediated artificial selection is another long-standing practice of red deer population management, both in the wild and on farms. In wild populations, the traditional hunting practice, largely for meat, is based on selective harvesting (negative selection) of non-fit animals predominantly. The exception was selective shooting for age class of trophy culmination in males/stags by trophy hunters. Over time, artificial selection has shifted more to trophy value and less to meat. However, classic positive genetic selection of males and estimated female breeding value for trophy value was only conducted in farms (as it is impossible to conduct in the wild). More recently, in game estates and deer parks, selected animals have also been transported and released into wild populations contained within fenced areas. In addition to the selection of national populations of deer, it is important to bear in mind the strong influence of New Zealand and British populations of deer, which, being the first to be selected for trophy in the last quarter of 20th century (see below), have been the source of genetic improvement for trophy (and meat, for their tameness) influencing crosses with national populations in farms of many European countries [11].

In wild populations, similar to translocation and introgression, artificial selection significantly affects the genetic variability and fitness of local autochthonous populations [9,22]. In these wild populations, artificial selection is predominantly now targeted to high-trophy animals, while dams with progeny are left untouched because they ensure population growth [23]. This could affect sex ratio, age structure and effective population size in addition to survival, reproduction and population growth. On the other hand, the effect of selective hunting pressure on the population gene pool is hard to predict because the population’s genetic variability could be affected by changes in population size and the migration of individuals, especially in wildlife [22].

While translocation and selective hunting have affected the genetic diversity of European deer populations for centuries, increasing land fragmentation (due to the fencing of highways, canals, and human settlements) accompanied by isolation of populations is now common [24]. A particular example of this phenomenon is the fencing of game estates and deer parks, which is considered the first management action in order to preserve the trophy quality and avoid the loss of valuable individuals [25]. Therefore, without monitoring of populations’ diversity and proper farm management (game estates and parks/game preserves), effective population size and genetic drift, and simultaneously increase of inbreeding in small and discontinuously distributed populations can be expected [1,9,26].

In the second half of the last century, farming of wild ungulates, especially cervids, became popular overseas, but also in western Europe. Although deer farming dates back possibly 2000 years, it was rather common in the middle age, at least in Britain (reviewed in Serrano et al. [11]), the first farm in the last century in Europe was established in 1970 in Scotland. Subsequently, due to the high demand for venison, particularly in central Europe outside the hunting season [11], farms were established in Germany on land unsuitable for agriculture. There, the surplus production of cereals, meat and milk contributed to the development of extensive farming, in order for the products to be market competitive. Subsequently, deer farms were created in other countries and are now widespread in most European countries. For example, in Belarus, the Czech Republic, Estonia, Latvia, Lithuania, Hungary, and Slovakia are farms and game parks with total land used cca. 80,000 ha and overall, 50,000 animals, predominantly red deer (*Cervus elephus* sp.), fallow deer (*Dama dama*) and European mouflon (*Ovis musimon*) [27]. Spain has the largest number of deer-managed in-game estates or deer parks, with over 2000 fenced game estates averaging 1000 ha (which adds up to 2.15 million ha with an estimated population of 650,000 deer [25]).

The first world exporter of deer venison, deer subproducts, and the number of farms and animals in a single country is New Zealand, with about one million farmed deer [11]. Therefore, New Zealand is recognised as a global leader in red deer farming and associated management (including genetic selection for a number of traits). In New Zealand, the first license for establishing a deer farm was awarded in 1970. The early history of red deer farming in New Zealand can be traced back to the late 19th and early 20th centuries when multiple introductions of animals, mainly from English parks and Scotland Highlands, were undertaken. The introduction of red deer to New Zealand was facilitated by the absence of native large browsing mammals in the country. The red deer found favourable conditions in the forests and grasslands of New Zealand, and without natural predators, their populations quickly grew. Today, red deer are an important part of New Zealand’s wildlife and farming landscape. They can be found in both wild populations and on deer farms, contributing to New Zealand’s hunting (trophy and velvet) and venison industries [11]. Moreover, the leading role of New Zealand in deer farming is supported by well-organised genetic research allowing for parentage testing, estimation of breeding values, and other genomic tests for traits of economic importance [28,29,30].

In sustainable agriculture, deer farming is considered a modern phenomenon that benefits both the country and its inhabitants. The use of marginal land to produce high-quality venison production can be regarded as the main advantage of deer farming. The development of deer farming in most European countries like Slovakia was slower than in New Zealand. Deer has radically different historical reasons for farming or harvesting in western and Asian cultures: for trophies (and secondary product its venison) in western culture, whereas in China and the rest of Asia has been used for 2000 years in their traditional medicine, mainly growing antler (also called velvet). Only New Zealand, Russia and former Soviet countries like Kazakhstan, and to a lesser extent, Argentina, farm deer mainly for velvet or in addition to venison [11]. As an example for the European Union, in Slovakia, similarly to other countries, an organisation representing deer farmers (Slovak Association of Deer Farming, SADF) was established in 2008.

Therefore, human activities are expected to have left indelible traces in the genomes of farmed as well as wild deer. In this study, we aimed to analyse the gene pool of two wild and seven farmed red deer populations to test the effect of human-mediated selection on their genome structure and quantify potential gene flow between them using genotyping-by-sequencing (GBS) data.

## 2. Materials and Methods

### 2.1. Animals Collection and Data Mining

In cooperation with the Slovak Association of Deer Farming, 138 farmed deer hair root samples from seven European countries, with some of the animals originating from New Zealand, were collected by plucking 50–100 hairs and storing them in a dry environment. Subsequently, the database was supplemented with 164 animal tissue samples from the wild, representing the gene pool of Carpathian (formerly *Cervus elaphus elaphus*) and Spanish red deer (formerly *Cervus elaphus hispanicus*) (Table 1). When we refer to the New Zealand red deer population subsequently, we also implicitly acknowledge that it is derived composite strain of primarily English and Scottish origins. After nuclear DNA extraction using an initial protease K digestion from salt and alcohol precipitation [31], the genome of animals was analysed by the genotyping-by-sequencing (GBS) method. The GBS libraries were prepared using the *Pst*I enzyme following Elshire et al. [32] and Dodds et al. [33]. The nuclear DNA of each animal was sequenced in cooperation with the AgResearch (Mosgiel, New Zealand) by Illumina HiSeq 2500 using 101bp single-end sequencing and version 4 chemistry. After DNA sequencing, all low-quality reads (average phred33 score below 30) were filtered out using the trimmomatic v0.39 program [34].

SNP calling for GBS data was conducted by Tassel v3.0.174 [35] based on the UNEAK pipeline (without the use of a reference genome). The following criteria were used: *-UFastqToTagCountPlugin -c 1 -e PstI*; *-UMergeTaxaTagCountPlugin -c 3*; *-UTagCountToTagPairPlugin -e 0.03*; *-UTagPairToTBTPlugin*; -*UMapInfoToHapMapPlugin -mnMAF 0.01 -mxMAF 0.5*. We identified initially 69,379 SNPs with an average depth of reads across SNPs and samples of 3.144 and call rate of 0.655. Genotype information obtained was then transformed using VCFtools v3.0 [36] to the input files for PLINK v1.9 [37]. Finally, the quality control (QC) of data was performed to select individual SNPs with a minimum call rate of 0.8 and a minimum minor allele frequency (MAF) of 0.01. Overall, 28,116 SNPs and 302 individuals passed the QC criteria.

### 2.2. Overall Heterozygosity

Even though red deer populations under study belong to the same species, breeding goals and selection criteria could differ depending on the country of origin. In this study, the effect of human activity on the gene pool of wild as well as farmed red deer populations was derived from the level of observed and expected heterozygosity and genomic inbreeding. The applied approach assumed that processes such as translocation, introgression and selective breeding significantly changed the intrapopulation level of overall heterozygosity.

For the calculation of observed (*H_o_*) and expected heterozygosity (*H_e_*) and genomic inbreeding (*F_HOM_*), PLINK v1.9 [37] was used. *F_HOM_* was derived from the genome-wide homozygous excess according to the formula:

(1) $$F_{HOM}=\frac{O-E}{N-E},$$

where *O* is observed homozygous genotype counts for each individual, *E* is expected homozygous genotype counts under the Hardy–Weinberg equilibrium (HWE) for each individual and *N* is the total number of genotypes.

### 2.3. Population Structure

The degree of population stratification and genetic relationships within and among analysed populations were tested based on the calculation of the *F_ST_* fixation index [38], Nei’s genetic distances [39], using discriminant analysis of principal components (DAPC) [40] and by superparamagnetic clustering approach [41].

The *F_ST_* index is usually used to estimate the degree of genetic differentiation among populations and quantify genetic relationships between them. This index is generally considered a good indicator of the intensity of population fragmentation expressed by a decrease in heterozygosity in subpopulations due to genetic drift. The *F_ST_* index can be defined as follows [38]:

(2) $$F_{ST}=\frac{H_{T}-H_{S}}{H_{T}},$$

where *H_T_* is the expected heterozygosity in metapopulation, and *H_S_* is the average expected heterozygosity in subpopulations. The final *F_ST_* matrix supported by 1000 bootstrap replications was constructed using the R package StAMPP [42].

Nei’s genetic distance theory assumes that if two populations with low genetic distances are similar, they have most likely common ancestors. When considering two populations, *X* and *Y*, where *x_i_* and *y_i_* are the frequencies of the *i-th* allele in populations *X* and *Y*, the probability of matching two randomly selected genes is $j_{x}=\sum x_{i}^{2}$ in populations *X* and $j_{y}=\sum y_{i}^{2}$ in population *Y*. The probability of matching two randomly selected genes, one from each population, is then $j_{xy}=\sum {x_{i}y}_{i}^{2}$. According to this, Nei’s standard genetic distance can be calculated as [39]:

(3) $$D=-\ln I;\;I=\frac{J_{xy}}{\sqrt{J_{x}J_{y}}},$$

where *I* is the normalised gene identity (or genetic identity) between the *X* and *Y* populations. *J_x_*, *J_y_,* and *J_xy_* are the arithmetic mean of *j_x_*, *j_y_,* and *j_xy_* over all loci in the genome, including monomorphic ones. Nei’s genetic distances within and between populations were calculated using the R package StAMPP [42].

Genetic variance distributed within and between analysed populations was quantified by discriminant analysis of principal components using the R package Adegenet v2.1.3 [40]. This multivariate method, which is used to identify and describe clusters composed of genetically similar individuals, allows a visual assessment of intrapopulation differentiation, determining the share of individual alleles in the population structure and the membership probability of each individual. DAPC was based on predefined groups, reflecting the origin of the analysed individuals. In the first step, the input genotype data were transformed by principal component analysis (PCA) into uncorrelated variables corresponding to the total variance stored in the dataset. Subsequently, these uncorrelated variables were used to maximise the estimate of variance between groups by determining discriminant functions (DF), representing a linear combination of original variables (alleles) with the highest possible variance between groups and the lowest within them. The optimum number of principal components corresponding to the highest proportion of variance in the dataset was tested using α-score calculation [43].

The degree of differentiation within and between populations was then tested through unsupervised cluster analysis, also called superparamagnetic clustering (SPC) [41]. In the first step, a symmetric matrix of IBD distances between individuals was constructed using PLINK v1.9 [37]. Subsequently, the obtained matrix was analysed and graphically visualised using the Netview package [44]. Finally, the optimal number of clusters was tested by calculating the k-NN value, expressing the maximum number of interconnected individuals.

### 2.4. Genetic Admixture and Gene Flow

The degree of genetic admixture between populations was quantified based on the Bayesian approach implemented in Structure v2.3.6 [45]. The analysis was performed based on the default parameters of the admixture model and the correlations between allele frequencies using 10,000 burn-in periods and 100,000 MCMC (Markov chain Monte Carlo) replications. The tested number of clusters (K) ranged from 1 to 20. Each run was repeated 20 times. The optimum K value was determined using the web-based tool Structure Harvester [46]. Subsequently, the effect of gene flow between populations on their genetic composition was evaluated by estimating the migration rate between populations using an assignment test in Bayesass v1.3 supported by 1000 iterations [47].

## 3. Results

### 3.1. Overall Heterozygosity

Generally, the average observed and expected heterozygosity was relatively low in all analysed populations of red deer (Table 2). Observed heterozygosity ranged from 0.072 (Spanish populations) to 0.182 (Slovak wild population). The expected heterozygosity showed a similar trend as observed (0.182–0.154). A comparison of both parameters showed that *H_e_* values were higher than the observed heterozygosity. When the expected heterozygosity values are higher than observed, the populations are assumed to deviate from the Hardy–Weinberg equilibrium and, in that case, they can be significantly affected by factors such as inbreeding, migration, selection, or genetic drift. French and Slovak wild populations showed the lowest differences between observed and expected heterozygosity, which suggests that the bottleneck effect (founder effect in farmed animals) had a minor effect on the population gene pool. On the contrary, the highest difference between observed and expected heterozygosity was found in the Hungarian and Spanish populations.

The MAF level observed across animals and SNPs within each analysed population corresponds to the obtained level of heterozygosity (Table 2). The Spanish population showed the lowest MAF level (0.105), whereas the highest average value of MAF was found in the Slovak population (0.122). The overall average MAF was 0.15 with a confidence interval (95%) of 0.15–0.16.

Genomic inbreeding derived from the observed and expected homozygous genotype counts ranged from 0.035 (Slovak wild population) to 0.359 (Spanish populations) (Table 2). Similar to MAF, the level of genomic inbreeding reflects the level of heterozygosity within the gene pool of each red deer population.

### 3.2. Population Structure

In the first step, two commonly accepted parameters were used to quantify genetic relationships between populations, Nei’s genetic distances and *F_ST_* fixation index (Table 3). The average value of the *F_ST_* index (0.077 ± 0.033) suggested a certain degree of genetic connectedness among the analysed red deer populations, which subsequently confirmed the Nei’s genetic distances (in average 0.025 ± 0.006).

As expected, because of the origin of populations, the highest genetic distance derived from the *F_ST_* index was observed between the Spanish population and the French (*F_ST_* = 0.141), Slovak (*F_ST_* = 0.134) or New Zealand populations (*F_ST_* = 0.138). A similar genetic distance was observed between the wild Slovak population and either the New Zealand (*F_ST_* = 0.125) and the French population (*F_ST_* = 0.112). On the contrary, very low genetic distances were observed between the German, Latvian, and Polish farmed populations (*F_ST_* in the range of 0.020–0.027). Relatively low genetic distance was also found between the Slovak farmed and the Hungarian farmed population (*F_ST_* = 0.015).

In the case of Nei’s genetic distances, the Spanish population also showed the highest degree of genetic differentiation from others (*D_A_* in the range of 0.027–0.039). Similar to the *F_ST_* index, the second population showing a high genetic distance from other populations in the analysis was the Slovak wild populations, which revealed a relatively high degree of differentiation, mainly in the case of farmed populations from France and New Zealand (*D_A_* = 0.035 resp. *D_A_* = 0.036). Matrices of Nei’s genetic distances confirmed that Slovak farmed and Hungarian populations are genetically closely connected with *D_A_* value of 0.013. A similar level of genetic distances was also found between populations from Germany, Poland, and Latvia (*D_A_* in the range of 0.013–0.014).

**Figure 1:**
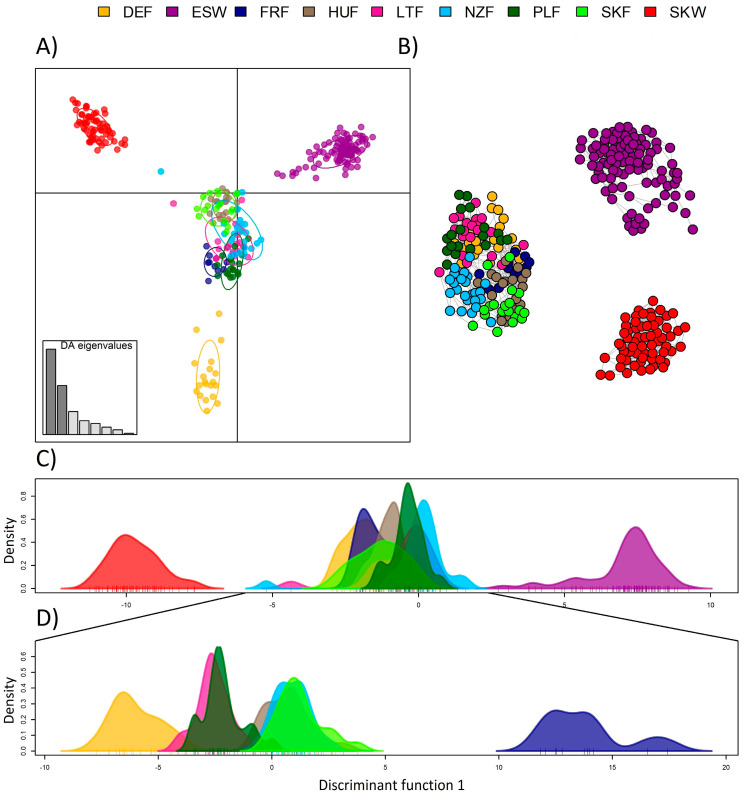
Interconnection among analysed populations resulting from DAPC (**A**), unsupervised clustering method based on genetic distances (**B**), the first discriminant function of DAPC (**C**), and first discriminant function of DAPC without Slovak and Spain populations (**D**) (DEF—German farmed, ESW—Spanish wild, FRF—French farmed, HUF—Hungarian farmed, LTF—Latvian farmed, NZF—New Zealand farmed, PLF—Polish farmed, SKF—Slovak farmed, and SKW—Slovak wild).

In the second step, the genetic fragmentation of analysed populations was tested using DAPC and unsupervised cluster analysis implemented in the R package Netview [44]. Figure 1 illustrates the representative results resulting from both applied approaches. The first and second discriminant functions of DAPC (Figure 1A) and the unsupervised clustering approach (Figure 1B) clearly separated the Slovak wild (in red colour) and Spanish (in purple colour) populations to separate clusters. In addition, the results indicated a partial differentiation of the German population (in yellow colour) from other farmed populations in the analysis. However, Figure 1C showing results based on the first discriminant function, indicated that farmed populations formed one joint genetic cluster. Due to this, DAPC was performed separately only for farmed populations (Figure 1D). This analysis confirmed previous results, indicating significant differences in the gene pool between the French and other European farmed populations and partial differentiation of the German population. Consistent with Nei’s genetic distances and *F_ST_* index, the Polish, Latvian, and German populations showed a relatively high level of genetic connectedness. Another partially separated genetic group was formed by the Slovak farmed, New Zealand, and Hungarian populations.

### 3.3. Genetic Admixture and Gene Flow

**Figure 2:**
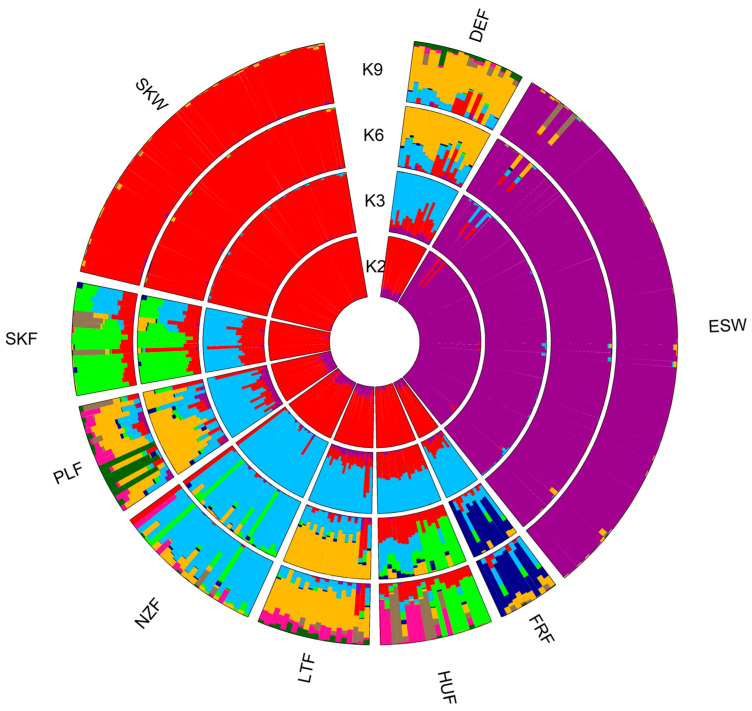
Stacked circular bar plots representing the cluster membership probabilities (K = 2, K = 3, K = 6, K = 9) suggested by the Structure algorithm. Colours represent the degree of admixture among populations for each K (DEF—German farmed, ESW—Spanish wild, FRF—French farmed, HUF—Hungarian farmed, LTF—Latvian farmed, NZF—New Zealand farmed, PLF—Polish farmed, SKF—Slovak farmed, and SKW—Slovak wild).

The Bayesian approach implemented in Structure v2.3.6 [45] and Bayesass v1.3 [47] was used to analyse the degree of genetic admixture and intensity of gene flow between populations. Figure 2 shows representative results of admixture analysis for K2, K3, K6, and K9. According to the ΔK, the optimal number of clusters was three, corresponding to the two wild populations (Slovak and Spanish) and the group of farmed populations.

Comparison of admixture proportion within the gene pool of analysed populations showed that the genetic divergence of wild Slovak and Spanish populations from others resulted in a very low degree of shared genetic variants between them, i.e., very low level of admixture. On the other hand, farmed red deer populations showed a relatively high level of genetic admixture compared to wild populations. In the case of farmed animals, the lowest proportion of admixture was found in the gene pool of French and New Zealand populations. However, all analysed farmed populations shared a certain proportion of genetic variants coming from the New Zealand gene pool (Figure 2 K6 and K9). The New Zealand genetics could mainly affect the Slovak, German, Polish, and Latvian gene pool. In addition, populations from Germany, Poland, and Latvia shared common genetic variants that pointed to gene flow between them. As expected, the genetic connectedness to the Carpathian red deer (here, represented by Slovak wild population) was partly evident only in the gene pool of Hungarian and Slovak farmed populations, showing at the same time a high degree of genetic similarity between each other.

**Figure 3:**
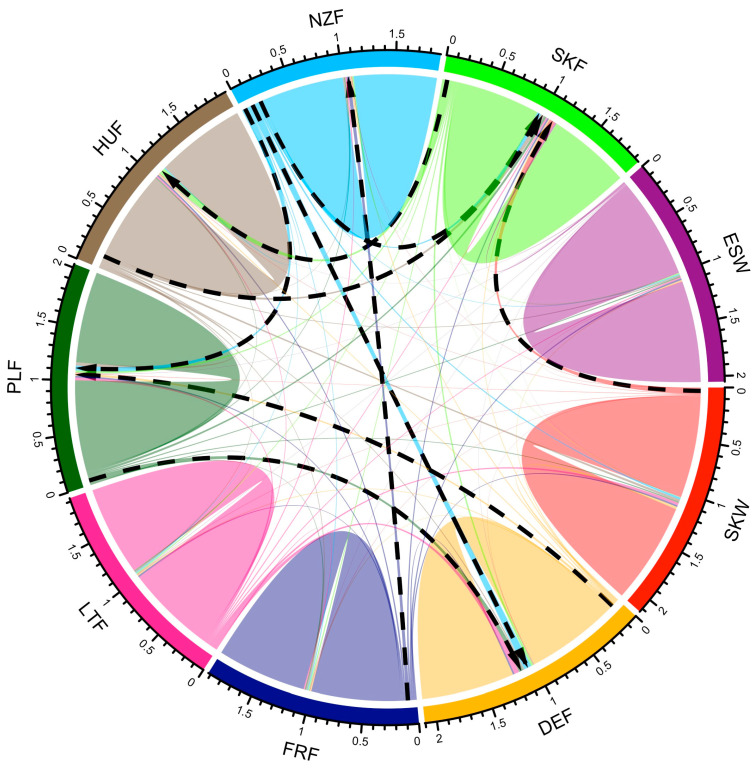
Relative migration rate between tested populations of red deer. The black dashed lines with arrows indicated the most intense rate of migration and its direction (DEF—German farmed, ESW—Spanish wild, FRF—French farmed, HUF—Hungarian farmed, LTF—Latvian farmed, NZF—New Zealand farmed, PLF—Polish farmed, SKF—Slovak farmed, and SKW—Slovak wild).

The relative migration rate among analysed populations is illustrated in Figure 3, where the black dashed lines with arrows indicate the most intense migration rate and its direction. Relative migration rate ranged from 0.003 to 0.073 with an average of 0.016 ±0.014. Because of different geographical origins, the Spanish population showed the lowest relative migration rates concerning other populations (on average 0.005). The higher relative migration rate of the wild Slovak population compared to the Spanish (on average 0.011) resulted from the fact that Slovak wild animals were used as founders in the case of the Slovak farmed population. The highest level of relative migration rate was found between New Zealand and German populations (0.073), Hungarian and Slovak farmed populations (0.064), and New Zealand and Slovak farmed populations (0.051).

## 4. Discussion

### 4.1. Data Mining

Genotyping-by-sequencing (GBS) used to discover SNPs in the genome of analysed red deer populations belongs to the group of genotyping methods that utilise next-generation sequencing (NGS) technologies. GBS is a simplified reduced-representation sequencing approach similar to RAD sequencing that is popular in animal and plant genetics as a low-cost alternative to whole genome sequencing and SNP genotyping microarrays [48,49,50,51]. As previous studies have shown, GBS can be successfully applied in population genetics [51], animal breeding and genomic selection [29,52] or genome-wide association studies [53]. On the other hand, low-coverage sequencing approaches, including GBS, may produce missing data and cause under-calling of heterozygotes due to the limited number of reads and uneven coverage capturing information for only one allele in a heterozygote, causing its misidentification as a homozygote [54,55]. Obtained average read depth across SNPs and samples and call rate are similar to previous studies in livestock [53,56] and wild animal species [30,57,58].

The removal of markers that do not conform to Hardy–Weinberg equilibrium expectations is commonly used as part of quality control in population-genetic studies. However, it was not applied in this study. The hypothesis we tested assumes that due to human-mediated selection as well as other factors, a change in allele frequencies can be observed in the genome of the tested farmed red deer populations compared to wild and thus they do not necessarily exhibit Hardy–Weinberg equilibrium. Wild animal species often exhibit population substructure or genetic differentiation due to factors like geographic barriers, limited gene flow, or local adaptation and varying levels of inbreeding due to small population sizes or social structures. These factors can violate the assumptions of Hardy–Weinberg equilibrium, resulting in departures from equilibrium even in the absence of genotyping errors. It was showed that departure from HWE with excess of heterozygosity is suggestive for genotyping errors and, on the other side, loss of heterozygosity pointed to natural forces such as population substructure [59]. Pearman et al. [60] showed through analyses of in silico and empirical datasets that some of the most widely used HWE filtering approaches dramatically impact inference of population structure. Moreover, SNPs out of HWE could be beneficial in specific scenarios but should be treated more carefully [61].

### 4.2. Overall Heterozygosity

One of the factors that may have contributed to the reduction of heterozygosity in analysed populations was the bottleneck effect (in farmed populations founder effect) because farmed populations generally arise from a limited number of founders. In addition, most breeders buy animals from the best or high-quality farms that bought from the best, thus further increasing the bottleneck effect. However, the bottleneck effect also affects wild populations, mainly due to anthropogenic factors. During the 20th century, the diversity of all wild red deer populations was affected by the 1st and 2nd World Wars, which led to a drastic decline in the population size. Although some populations were later restored with what remained from genetic diversity, their gene pool was affected by intensifying agricultural activity and urbanisation. The lowest observed heterozygosity was found in the deer population from Spain. This is most likely the results of two facts: 1) the human pressure for use of land and need of hunting to improve the diet left at the beginning of 20th century only three populations in Spain [62]: central (mount of Toledo), west (Extremadura), and one spot in the south (Sierra Morena), a situation that was aggravated during the food crisis derived from the Spanish civil war; 2) re-populations from these and from the few game farms existing in the last 30 years of fast growth of new game estates (pers. comm.). A similar low level of heterozygosity due to inbreeding has been found in Pyrenean chamois and wolves [63,64]. On the other hand, the highest level of observed heterozygosity was found in the Slovak wild red deer population. Based on this, it can be assumed that the gene pool of the wild Slovak population preserves the highest level of genetic diversity among the tested populations.

An important factor that indicates the effect of selection, migration, or genetic drift on the population genetic structure is the difference between the observed and expected heterozygosity, i.e., the deviation from the Hardy–Weinberg equilibrium. From analysed populations, significant deviations were found for the Hungarian and Spanish populations. Especially in the case of the Spanish population, it is likely to be the result of the bottlenecks mentioned above, or else, its geographical isolation from other local deer populations under study. In the Hungarian population, heterozygosity may have been affected mainly by the intensive selection of animals for trophy quality (which is also the case of privately managed wild deer populations in game estates such as that of the Spanish samples).

It was shown that the usability of genetic markers in population genetic studies depends on their polymorphic nature, i.e., level of minor allele frequency [65]. The overall average MAF was comparable to other studies related to quantifying genetic diversity in non-model species [66,67,68]. Except for the Spanish population, MAF values were relatively uniform across red deer populations under study (Table 2). In addition, observed MAF values correspond to the observed and expected heterozygosity. Thus, assuming a low MAF, a reduction in the proportion of overall heterozygosity in the gene pool of the population can be expected.

As expected, based on the level of observed and expected heterozygosity, the Slovak wild population showed the lowest level of genomic inbreeding (*F_HOM_* = 0.035), in this study derived from the observed and expected homozygous genotype counts. On the other hand, the observed excess of homozygous genotypes proportion in the Spanish population gene pool is probably the result of the historical bottlenecks mentioned above or its geographical isolation and the unbalanced effect of founders; thus, obtained *F_HOM_* value represents mainly the historical load of inbreeding. The founder effect was also fully reflected in the gene pool of farmed red deer populations, with an average *F_HOM_* from 0.04 to 0.30. Similar levels of *F_HOM_* have also been found in other livestock and companion animals showing at the same time a high level of intra-population homozygosity, e.g., local dog breeds [69], Wagyu cattle [70], and Kladruber horses [71]. Despite the high levels of genomic inbreeding, there was a minor deleterious effect of relatives mating because of the genetic “cleaning” over time. Although not previously described, it seems to be a logical explanation of the obtained high rate of genomic inbreeding in wild and farmed red deer populations. In the case of red deer, natural, and human-mediated negative selection can be considered a form of genetic purification. Thus, the anthropogenic factors affected the diversity of tested populations both directly through artificial selection (positive in farmed animals and negative in wild populations) and at the same time indirectly as a factor influencing the environmental conditions of local populations.

The reliability of the estimation of parameters quantifying heterozygosity of populations and differences between them could have been affected by the unequal number of samples per population. Nevertheless, the high standard deviation of the diversity parameters resulting from the genetic differences between individuals in tested populations points to a relatively low level of genetic relatedness between analysed animals that probably do not have a common ancestor and, therefore, reliably represent the gene pool of tested populations.

### 4.3. Population Structure

All applied approaches demonstrated that even if analysed red deer populations are genetically connected, they create separate units. This corresponds to the fact that the gene pool of European red deer populations has been affected for many centuries by both natural changes and anthropogenic activity depending on their geographical distribution [1]. Because red deer populations are widespread in different habitats, the frequencies of their genes may have changed locally due to natural selection and adaptation to different environmental conditions. At the same time, there may have been an increase in genetic admixture due to agricultural land use and the effects of climate change [10].

Both Nei’s and *F_ST_* matrices indicated the Spanish population as a genetically most distant group from others in the analysis (Table 3). This result is logical because this population has different geographical and phylogenetic origins, i.e., the gene pool. Furthermore, a relatively high degree of differentiation was found between the Slovak wild and farmed populations from France and New Zealand. On the contrary, the Slovak and Hungarian farmed populations demonstrated a relatively high degree of genetic similarity. Thus, the results indicate that these populations are genetically connected, probably due to the recent intensive exchange of genetic material. In addition, observed results pointed to historical connectedness between farms in the Baltic region of Germany, Poland, and Latvia. On the other hand, deer farms in France seem to have developed in part independently of other farms in Europe.

The results of DAPC and unsupervised cluster analysis confirmed assumptions resulting from Nei’s and *F_ST_* matrices. Both wild red deer populations formed separate genetic units differentiated from farmed animals (Figure 1). On the other hand, farmed populations created a single cluster, probably because of a certain proportion of common alleles shared in their gene pool. However, a closer look at the farmed populations confirmed that even if they are genetically closely connected, they vary in genetic composition (Figure 1D). As expected, the French population was separated from others. Common genetic clusters formed Polish, German, and Latvian populations and Slovak farmed, New Zealand and Hungarian populations. This indicates that both in the distant past as well as more recently, gene exchange has probably occurred among populations within clusters. In the case of Slovak farmed population, farmers have imported maternal genetics from Hungary and paternal genetics from New Zealand, which could explain their genetic similarity. It is important to mention in this point that the first insemination station for deer, mouflons and fallow deer in the European Union was established in Slovakia.

Based on different phenotypic traits, several subspecies of red deer have been historically distinguished. However, their taxonomic classification is still debated because the classification itself is often inconsistent with the results of genetic studies [72]. Due to its importance and distribution, the red deer has been the subject of several studies focused on the evolutionary history of populations living in different geographical locations [73]. Mitochondrial DNA-based studies have shown that the degree of genetic diversity of red deer populations in Europe reflects its phylogeographical origins [74]. Several studies have described three main mitochondrial lineages resulting from the genetic variability of the mtDNA D-loop region: the Western European lineage, the Eastern European lineage spread in the Balkans and the Mediterranean lineage that originated from Africa, Sardinia, and Corsica. Despite attempts to support a different subspecies for *C. e. hispanicus* based on nuclear microsatellite DNA instead of the most appropriate mitochondrial one [6], the three genetic lineage patterns (although not achieving enough divergence to differ at the subspecies level) were also found in several other studies based on nuclear microsatellites ([7]; even despite being blurred by recent translocations [75]).

### 4.4. Genetic Admixture and Gene Flow

The results of the DAPC showed a high degree of genetic connectedness between analysed farmed red deer populations. Increasing profits often leads deer breeders to buy breeding animals from abroad, thus exchanging genetic material between countries. Genetic differences between countries create a precondition for accelerating genetic progress, e.g., through the heterosis effect [76]. Because of this, it is important to quantify the intensity of gene flow and the degree of genetic admixture between populations.

Slovak wild and Spanish populations showed only a low level of genetic admixture, which confirms their phenotypic and genotypic divergence from other populations under study. Based on the DAPC results, we assumed that the group of farmed populations would be divided into four genetic clusters: first, composed of German populations, second, composed of the French population, third, consisting of Slovak farmed, Hungarian, and New Zealand populations and the fourth consisted of populations from Poland and Latvia. However, the observed admixture proportion only partially confirmed this assumption. Compared to previous results, traces of genetic variants coming from the New Zealand population were observed in the gene pool of all evaluated farmed populations. The highest proportion of these variants was found in the gene pool of Slovak farmed, German, Polish, and Latvian populations. The observed level of gene flow between populations was consistent with the admixture proportion; thus, identified traces of genetic admixture in the gene pool of farmed populations reflected a strong contemporary migration rate among them.

Previous studies were mostly oriented to the analysis of red deer populations spread across a particular country [77,78,79], but the results of this study confirmed that the effect of human-mediated selection should be considered as one of the most important factors affecting the level of admixture and gene flow in farmed red deer populations in a global view.

## 5. Conclusions

This study suggests that although the history of deer farming is relatively short compared to other traditional livestock species, it may leave significant traces in their gene pool. All of the approaches used indicated that intensive gene flow within farmed red deer populations has led to significant changes in allele frequencies compared to the wild populations. Due to higher levels of genetic diversity and homogeneity between sampling areas in farms, the genetic changes caused by human-mediated selection can be evolved much faster than in wild populations, even being subjected to the negative selective force of hunting management. It is also the first transnational evaluation of genetic diversity in red deer that has used a sequenced-based discovery and genotyping approach comprising a large number of DNA variants, which is not subject to ascertainment bias effects common in alternative approaches.

## Figures and Tables

**Table 1:** Description of the red deer populations examined.

Population		Abbreviation	No. of Animals
Germany	farmed	DEF	20
Spain	wild	ESW	102
France	farmed	FRF	11
Hungary	farmed	HUF	20
Latvia	farmed	LTF	20
New Zealand	farmed	NZF	27
Poland	farmed	PLF	20
Slovakia	farmed	SKF	20
Slovakia	wild	SKW	62

**Table 2:** Diversity indices showing heterozygosity level in analysed populations.

Population	*H_O_* ± SD (SE)	*H_e_* ± SD (SE)	MAF ± SD (SE)	*F_HOM_* ± SD (SE)
DEF	0.145 ± 0.158 (0.001)	0.176 ± 0.162 (0.001)	0.119 ± 0.131 (0.001)	0.127 ± 0.211 (0.047)
ESW	0.072 ± 0.102 (0.001)	0.154 ± 0.168 (0.001)	0.105 ± 0.133 (0.001)	0.359 ± 0.111 (0.011)
FRF	0.151 ± 0.197 (0.001)	0.158 ± 0.177 (0.001)	0.113 ± 0.144 (0.001)	0.036 ± 0.139 (0.042)
HUF	0.097 ± 0.128 (0.001)	0.169 ± 0.166 (0.001)	0.115 ± 0.132 (0.001)	0.295 ± 0.177 (0.040)
LTF	0.111 ± 0.137 (0.001)	0.171 ± 0.164 (0.001)	0.116 ± 0.132 (0.001)	0.242 ± 0.183 (0.041)
NZF	0.114 ± 0.147 (0.001)	0.165 ± 0.176 (0.001)	0.116 ± 0.142 (0.001)	0.242 ± 0.226 (0.043)
PLF	0.119 ± 0.143 (0.001)	0.170 ± 0.165 (0.001)	0.116 ± 0.132 (0.001)	0.219 ± 0.223 (0.050)
SKF	0.114 ± 0.147 (0.001)	0.166 ± 0.167 (0.001)	0.114 ± 0.134 (0.001)	0.253 ± 0.226 (0.046)
SKW	0.182 ± 0.158 (0.001)	0.187 ± 0.143 (0.001)	0.122 ± 0.118 (0.001)	0.035 ± 0.069 (0.009)

DEF—German farmed, ESW—Spanish wild, FRF—French farmed, HUF—Hungarian farmed, LTF—Latvian farmed, NZF—New Zealand farmed, PLF—Polish farmed, SKF—Slovak farmed, SKW—Slovak wild, *H_o_*—observed heterozygosity, *H_e_*—expected heterozygosity, MAF—minor allele frequency, *F_HOM_*—genomic inbreeding, SD—standard deviation, SE—standard error.

**Table 3:** Genetic relationships among populations based on Nei’s distances (under the diagonal) and *F_ST_* index (above the diagonal).

	DEF	ESW	FRF	HUF	LTF	NZF	PLF	SKF	SKW
DEF		0.103	0.076	0.050	0.025	0.058	0.027	0.066	0.074
ESW	0.027		0.141	0.119	0.101	0.138	0.101	0.134	0.121
FRF	0.027	0.039		0.085	0.072	0.087	0.074	0.099	0.112
HUF	0.020	0.032	0.031		0.045	0.065	0.047	0.015	0.061
LTF	0.014	0.027	0.027	0.020		0.055	0.020	0.062	0.074
NZF	0.020	0.035	0.029	0.023	0.020		0.053	0.069	0.125
PLF	0.014	0.027	0.027	0.020	0.013	0.019		0.064	0.080
SKF	0.023	0.035	0.033	0.013	0.023	0.023	0.023		0.074
SKW	0.023	0.030	0.035	0.020	0.023	0.036	0.025	0.023	

DEF—German farmed, ESW—Spanish wild, FRF—French farmed, HUF—Hungarian farmed, LTF—Latvian farmed, NZF—New Zealand farmed, PLF—Polish farmed, SKF—Slovak farmed, SKW—Slovak wild.

## Data Availability

The data presented in this study are available on request from the corresponding author. The data are not publicly available due to ongoing research.
